# Extensive Deep Vein Thrombosis in a Young Man Taking Tirzepatide for Weight Loss

**DOI:** 10.1016/j.aace.2024.08.011

**Published:** 2024-09-05

**Authors:** Mohammed Fareeduddin Farooqi, Muhammad Arshad Mehmood, Maria Khan, Hafiz Muhammad Salman, Adnan Agha

**Affiliations:** 1 Department of Internal Medicine, Tawam Hospital, Al Ain, United Arab Emirates; 2 Pharmacy Department, Tawam Hospital, Al Ain, United Arab Emirates; 3 Department of Internal Medicine, College of Medicine & Health Sciences, United Arab Emirates University, Al Ain, United Arab Emirates

**Keywords:** tirzepatide, deep vein thrombosis, glucagon-like peptide-1 receptor agonist

## Abstract

**Background/Objective:**

Obesity and rapid weight loss are risk factors for developing deep vein thromboses (DVTs). Our aims were to present a patient who developed extensive DVT after relatively rapid and severe weight loss that followed taking tirzepatide and to raise the awareness among health care professionals regarding the risk of DVT that can be associated with significant weight loss due to these agents.

**Case Report:**

We present the case of a 20-year-old young man, with raised body mass index of >35 kg/m^2^, who was initiated on tirzepatide treatment for weight loss, with 12-kg weight lost over 6 weeks. The patient did not have any risk factors for thrombophilia including family history, any recent travel, immobilization, recent infections, or recent surgeries. He presented with left leg swelling, and physical examination revealed signs of proximal DVT, and ultrasound Doppler and computed tomography venography confirmed extensive left-sided DVT with complete obstruction of the common femoral and iliac veins. He underwent mechanical thrombectomy and was maintained on anticoagulation therapy. His investigations for thrombophilia screening excluded any other cause for DVT, with the etiology attributed to possibly rapid weight loss.

**Discussion:**

Newer and potent glucagon-like peptide 1 receptor agonists like tirzepatide are commonly used nowadays to induce weight loss in obese patients.

**Conclusion:**

Adequate risk assessments and close monitoring should be performed in patients initiating glucagon-like peptide 1 receptor agonists, particularly if they have risk factors for developing venous thromboembolism.

**Highlights**

• There is an increased risk of deep vein thromboses associated with obesity
• Glucagon-like peptide 1 receptor agonists can cause rapid weight loss
• Rapid weight can increase risk of deep vein thromboses
• Risk assessment should be performed before initiating these agents

**Clinical Relevance**

The incidence of obesity is increasing, and its treatment options including glucagon-like peptide 1 receptor agonists may be dispensed without medical indication or prescription, which may lead to serious side effects. Both obesity and rapid weight loss are related to increased risk of deep vein thromboses. This case report emphasizes the need for venous thromboembolism risk assessment in patients starting on glucagon-like peptide 1 receptor agonists.

## Introduction

Venous thromboembolism (VTE) affects approximately 5% of the population in their lifetime, manifesting as 2 primary conditions: (1) pulmonary embolism (PE) and (2) deep vein thrombosis (DVT). DVT constitutes the predominant presentation in nearly two thirds of VTE cases, and its incidence and prevalence have steadily increased worldwide in recent decades.^1^ The pathophysiology of VTE, along with the predisposing factors, aligns with the Virchow triad, encompassing venous stasis, vascular injury, and hypercoagulability.^2^ Common risk factors for DVT include local infection, external compression, trauma, medications such as estrogen-containing drugs, immobilization, recent surgery, obesity, and inherited conditions such as antiphospholipid syndrome, with more than 95% of DVT cases occurring in the lower limbs. Lower limb DVTs typically originate in the calf, with approximately three fourths resolving spontaneously; however, 25% progress to involve the proximal leg veins, where nearly half of these cases can embolize, leading to PE.^3^ Patients with identifiable risk factors for PE are labeled as provoked PE, whereas others are termed as unprovoked PE, and these patients may require further workup including testing for thrombophilia and/or screening for malignancy.^3^^,^^4^ Despite recent technologic advancements, PE remains the third most common cardiovascular disease globally and one of the leading causes of mortality and morbidity, with even higher mortality among the elderly and patients with associated comorbidities especially cancer, with a 1-year fatality rate after VTE approaching 23.0% in a recent study.^4^

Our case report describes of a young man who developed extensive left leg DVT after being initiated on glucagon-like peptide 1 receptor agonist (GLP1-RA) to facilitate weight loss.

## Case Report

A 20-year-old young man presented to the emergency department with a 3-day history of pain and swelling in his left leg. He described the pain as sharp, rating 7 out of 10 in intensity, with radiation to the thigh and back. He reported no history of trauma or any family history of thromboembolism. Recently, he had started Mounjaro (tirzepatide) on a private prescription, taking 7.5 mg weekly, with the intention of losing weight to meet the physical requirements for selection in security services. Over the past 3 weeks, he had lost 10 kg. His medical history included glucose-6-phosphate dehydrogenase deficiency and childhood asthma, for which he did not require any regular medication. He had been previously admitted several times for low back pain secondary to lumbar disc prolapse (confirmed by magnetic resonance imaging), managed with analgesia, resulting in complete resolution of symptoms.

Upon examination, he was in no apparent distress, and his vital signs including pulse rate and blood pressure were within normal limits. His chest was clear, and there were no cardiac murmurs heard. No palpable abdominal masses or abdominal tenderness was noted. His left thigh exhibited swelling, tenderness, and mild redness, with a left mid-thigh circumference of 33.5 cm compared with 30.5 cm (a difference of 3 cm) on the right side. Additionally, the left calf was swollen, measuring 2 cm larger than the right.

Lower limb venous Doppler studies confirmed extensive DVT involving the left common iliac, external iliac, common femoral, superficial femoral, popliteal, posterior tibial, and great saphenous veins (Fig.). Table 1 summarizes initial laboratory investigations of the patient. Other investigations for hepatitis B, hepatitis C, and viral hepatitis delta screen were all negative.

**Fig:**
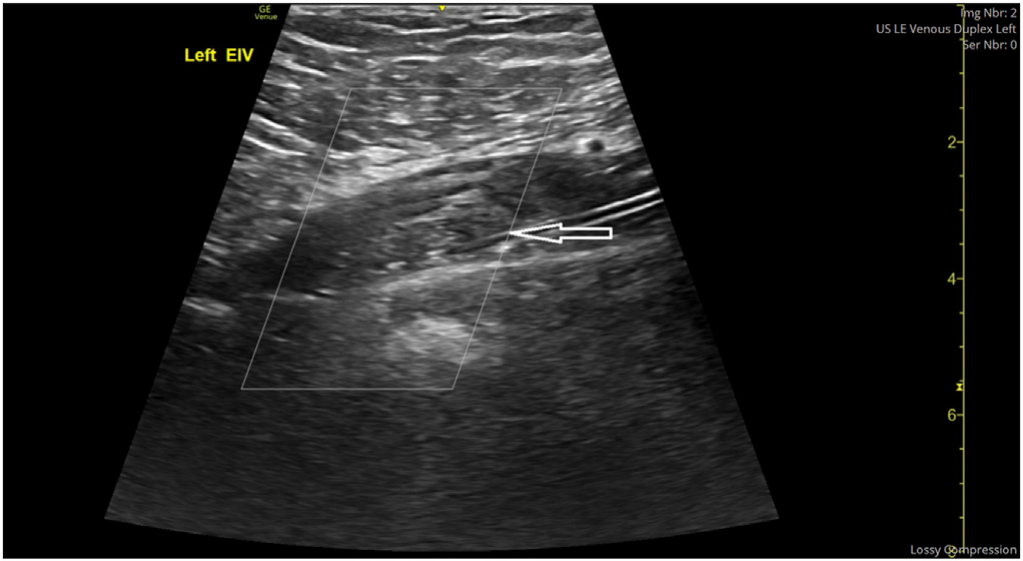
Left thigh ultrasound showing clot (white arrow) in the left external iliac vein. EIV, external iliac vein.

**Table 1:** Results of Patient Investigations

Investigations	Results	Normal range
Sodium	136 mmol/L	136-145 mmol/L
Potassium	3.4 mmol/L	3.2-5.5 mmol/L
Creatinine	51 μmol/L	62-106 μmol/L
Urea	2.83 mmol/L	2.80-8.10 mmol/L
Fasting glucose	4.9 mmol/L	3.9-5.5 mmol/L
Calcium corrected	2.37 mmol/L	2.10-2.60 mmol/L
Magnesium	0.73 mmol/L	0.66-1.07 mmol/L
Albumin	26 g/L	35-52 g/L
Total bilirubin	15 μmol/L	<21 μmol/L
Direct bilirubin	10 μmol/L	<5 μmol/L
Alkaline phosphatase	82 IU/L	40-129 IU/L
Aspartate aminotransferase	112 IU/L	<40 IU/L
Alanine aminotransferase	114 IU/L	<41 IU/L
Creatine kinase	403 IU/L	39-306 IU/L
Total cholesterol	4.5 mmol/L	3.90-5.20 mmol/L
LDL cholesterol	2.81 mmol/L	<2.59 mmol/L
HDL cholesterol	0.96 mmol/L	1.10-1.60 mmol/L
Triglycerides	1.67 mmol/L	0.50-1.70 mmol/L
HbA1c	4.5% (26 mmol/mol)	4.3%-5.6% (23-38 mmol/mol)
Probrain natriuretic peptide	20.7 ng/L	0-85 ng/L
Troponin	7.9 ng/L	<14 ng/L
White blood cell	8.4 × 10^9^/L	4.5-13 × 10^9^/L
Hemoglobin	11.5 g/dL	13.2-17.3 g/dL
MCV	78 fL	80-99 fL
MCH	26 pg	27-34 pg
Platelets	332 × 10^9^/L	140-400 × 10^9^/L
Prothrombin time	12.8 s	11-13.5 s
INR	1.15	<1.1
APTT	32 s	21-35 s
Fibrinogen level	1.18 g/L	1.50-3.8 g/L

Abbreviations: APTT = activated partial thromboplastin time; HbA1c = glycated hemoglobin; HDL = high-density lipoprotein; INR = international normalized ratio; LDL = low-density lipoprotein; MCH = mean corpuscular hemoglobin; MCV = mean corpuscular volume.

Because of the extensive thrombosis extending into the left common iliac vein, the vascular surgery team was consulted. The patient underwent thrombolysis via interventional radiology using alteplase followed by mechanical thrombectomy, resulting in the restoration of blood flow in the left common femoral and great saphenous vein. Echocardiography revealed normal findings without any evidence of right ventricular strain pattern, and blood cultures were negative. Anticoagulation therapy was initiated with subcutaneous low-molecular-weight heparin (enoxaparin 1 mg/kg twice daily) during hospitalization, subsequently switching to oral anticoagulation with edoxaban 60 mg once daily upon discharge. Given the unprovoked extensive DVT, further investigations including thrombophilia screening was sent during admission (results shown in Table 2). The patient was discharged and has remained symptom-free for 4 months on outpatient follow-up. The initial thrombophilia screening results were inconclusive, with anticardiolipin immunoglobulin G antibodies levels being elevated. The patient is scheduled for repeat thrombophilia testing follow-up with hematology outpatient clinic after completing 6 months of anticoagulation therapy.

**Table 2:** Results of Thrombophilia Screening of the Patient

**Investigations**	**Results**	**Normal range**
Factor V Leiden screen	0.96	0.18-1.13
Factor VIII	474%	50%-200%
Factor X	110%	70%-120%
Antithrombin III	90%	81.3%-133%
Protein C activity	136.6%	68.3%-143.5%
Free protein S	82	63-115
D-dimer	35 000 mg/L	0.124-0.523
Cardiolipin IgG	83.8 CU^a^	<20 CU
CRP	66 mg/L	<5 mg/L
B2 glycoprotein IgG	15.2 CU	<20 CU
B2 glycoprotein IgM	1.1 CU	<20 CV

Abbreviations: CRP = C-reactive protein; IgG = immunoglobulin G; IgM = immunoglobulin M.

a Raised cardiolipin immunoglobulin G antibodies may suggest a possible anti-phospholipid syndrome, however a single value is not diagnostic.

## Discussion

Obesity is one of the common risk factors associated with an increased risk of VTE; however, no evidence is available to suggest that subsequent weight loss can reduce this risk.^5^ The underlying mechanism remains incompletely understood. Similarly, findings from the Tromsø study indicate an increased risk of venous thromboembolism in participants who experienced weight loss, suggesting rapid fluctuation in weight as a potential risk factor.^6^ Another GLP1-RA, semaglutide, has been associated with a 266% (relative risk of 3.66) increased risk of VTE in a recent study, leading to concerns about its suitability for individuals at high risk of DVT.^7^ Other studies investigating GLP1-RAs have not reported any increased risk of DVT related to the use of these medications.^8^

Tirzepatide, a 39–amino acid modified peptide molecule, acts as a GLP1-RA and binds to gastric inhibitory polypeptide receptors, indicated for glycemic control and/or for chronic weight management in adults.^9^ Its central inhibitory action reduces appetite and food intake, making it a valuable therapy for weight loss. However, severe appetite suppression in some patients may lead to reduced oral intake and potential dehydration.^9^ The recent advancements and increased availability of GLP1-RA in the market have resulted in their widespread use, including by patients seeking weight loss, sometimes without thorough physician assessment. This trend can increase the incidence of gastrointestinal side effects such as diarrhea and the risk of dehydration. Despite both dehydration and morbid obesity being well-recognized risk factors for VTE, comprehensive evaluation for other VTE risk factors in these patients is often overlooked when initiating GLP1-RA treatment.

Our case serves to alert practitioners about the signs and symptoms of DVT that should be monitored upon starting GLP1-RA therapy. The precise mechanism responsible for the increased risk of DVT, whether attributed to rapid weight loss induced by these agents or dehydration resulting from gastrointestinal side effects, remains unclear. However, this uncertainty should be explained to patients, and a standard risk assessment, including past medical and family history of VTE and medication history, should be conducted before initiating GLP1-RA treatment. After starting GLP1-RA treatment, the patients who develop rapid weight loss should be monitored closely for side effects.

## Statement of Patient Consent

Informed consent was obtained from the patient ethical approval was taken from Tawam Hospital Human Research Ethics Committee with approval number MF2058-2023-993.

## Disclosure

The authors have no conflicts of interest to disclose.

## References

[1] Goldhaber S.Z. (2012). Venous thromboembolism: epidemiology and magnitude of the problem. Best Pract Res Clin Haematol.

[2] Stone J., Hangge P., Albadawi H. (2017). Deep vein thrombosis: pathogenesis, diagnosis, and medical management. Cardiovasc Diagn Ther.

[3] Olaf M., Cooney R. (2017). Deep venous thrombosis. Emerg Med Clin North Am.

[4] Tagalakis V., Patenaude V., Kahn S.R., Suissa S. (2013). Incidence of and mortality from venous thromboembolism in a real-world population: the Q-VTE study cohort. Am J Med.

[5] McLendon K., Goyal A., Attia M. (2024).

[6] Horvei L.D., Brækkan S.K., Hansen J.B. (2016). Weight change and risk of venous thromboembolism: the Tromsø study. PLoS One.

[7] Yin D.G., Ding L.L., Zhou H.R., Qiu M., Duan X.Y. (2021). Comprehensive analysis of the safety of semaglutide in type 2 diabetes: a meta-analysis of the SUSTAIN and PIONEER trials. Endocr J.

[8] Vosoughi K., Atieh J., Khanna L. (2021). Association of glucagon-like peptide 1 analogs and agonists administered for obesity with weight loss and adverse events: a systematic review and network meta-analysis. EClinicalMedicine.

[9] Farzam K., Patel P. (2024).

